# Supplementary material for: Dampened Slow Oscillation Connectivity Anticipates Amyloid Deposition in the PS2APP Mouse Model of Alzheimer’s Disease
Source: Cells. 2019 Dec 24;9(1):54. doi: 10.3390/cells9010054 (PMC7016892; doi:10.3390/cells9010054)
Supplement: Supplementary file 1 [file cells-09-00054-s001.pdf]

SUPPLEMENTARY MATERIALS

Power and Cross-Frequency-Coupling analysis

LFP power spectral density (PSD) was calculated for a 2.5-minute window through the Welch estimate method (pwelch.m) as described [1]. Hamming windows of 2 seconds were used with an overlap of 50%. The spectral power in the different frequency bands was obtained by computing the integral with the trapezoidal method (trapz.m) under the PSD curve, with frequency resolution of 0.01 Hz.

For the analysis of the Cross-Frequency-Coupling (CFC) index of SO (or delta) with higher bands, a 5-minute window was firstly Butterworth-filtered in the Slow and delta bands (3rd order filter) and then in the specific higher frequency band (6th order filter). Next, the Hilbert transform (hilbert.m) was applied to each time-series obtained from filtering. Then phase, angle and amplitude time-series were extracted from the Hilbert transform of SO and delta and the higher frequency filtering, respectively. Finally, PAC index was computed by means of the general linear model (GLM) as described in [2]. The GLM method was chosen owing to its sensitivity [3]. Basically, the amplitude of the faster oscillation is modelled by multiple regression and the index is the proportion of variance explained by the model.

Cross-correlation of instantaneous maximal LFP amplitudes

For the cross-correlation analysis we implemented previous methods [4]. The recording windows used for PAC analysis were band-pass filtered for the SO and delta bands, and the instantaneous amplitude of each signal was obtained from the Hilbert transform by a custom-written script. Subsequently, for all channels, maximal amplitude cross-correlation matrices were built with the Matlab function xcorr (xcorr.m) over latencies ranging from + 0.1 to − 0.1 s. The mean amplitude was first subtracted from each vector prior to cross-correlating, as the DC component of a signal has no relevance for cross-correlation. The latency (lag) at which the cross-correlation peaked was then determined. Matrices of maximal cross-correlation coefficients and associated lags were obtained for each mouse and then averaged by genotype and age-cohort.

Histology

The position of the LFP electrode insertion was sample checked. At the end of the experiment, the electrode was withdrawn, dipped in 1,1'-dioctadecyl-3,3,3',3'-tetramethylindocarbocyanine perchlorate (DiI) (0.05% in DMSO) and then carefully reinserted to the same stereotaxic coordinates of the mid depth. Next, the brain was dissected, and the left hemisphere was fixed in 4% PFA for 48 hours. After washing 3 times with TBS, the hemibrain was cut parallel to the coronal plane with a

vibratome (Leica VT1000 S), yielding 70-μm-thick slices. Finally, slices were stained with Hoechst 33342 (5 g/ml) in TBS for 20 minutes at room temperature (RT), washed 3 times in TBS and mounted by means of Mowiol mounting medium. The electrode insertion site was routinely visualized by epifluorescence microscopy as for immunohistochemistry.

Immunohistochemistry

Coronal brain slices (50 μm) were cut from the left hemisphere as described above and conserved in TBS at 4 °C until employed for dorsal hippocampus immunostaining. For staining, slices were selected over a range of 300 μm, close to the insertion site. Immunostaining was performed on floating slices. Slices were incubated in permeabilizing/blocking buffer containing 0.2% TritonX-100 and 5% goat serum in TBS for 1 hour at RT. Next, they were first incubated overnight at 4°C with rabbit anti-GFAP (Dako, 1:400), and then for 1 hour at RT in the dark, with the blocking buffer plus the Alexa488-conjugated donkey anti-rabbit (Invitrogen, 1:1000). Slices were finally incubated for 15 minutes with methoxy-X04 (0.01 mg/ml) in PBS at RT, mounted on microscope slides by means of a Mowiol, and stored at 4°C until visualization. Each step described above was separated from the next one by 3 washes in TBS (5 min each). Samples were imaged with a 4X objective by a TiE inverted microscope (Nikon), equipped with a Zyla 4.2 camera (Andor, Oxford Instruments). During acquisition, parameters for acquisition were kept constant.

ELISA

Hippocampi, dissected from the right brain hemispheres, were immediately snap-frozen in liquid nitrogen and stored at -80°C until processing. Aβ was extracted by a 2% sodium dodecyl-sulphate (SDS)- radio-immunoprecipitation assay (RIPA) buffer (50 mM Tris, 150 mM NaCl, 2% sodium deoxycholate, 1% Nonidet P-40) also containing protease and phosphatase inhibitors (cOmplete, Mini, protease inhibitor cocktail, Roche; PhosSTOP in RIPA buffer phosphatase inhibitor cocktail, Roche) and secondly by 70% formic acid (FA) as previously described [5]. At the moment of performing the enzyme-linked immunosorbent assay (ELISA), the RIPA fraction was diluted 1:10 in the assay standard diluent while the FA fraction was centrifuged and diluted 1:20 in the neutralization buffer (1 M Tris, 0.1 M NaCl, 0.025% phenol red pH 7) before being assayed in triplicate for each mouse (n=3 each tg lines). For measuring Aβ42 levels, we used the Human/Rat Beta Amyloid (42) 290-62601 and High-sensitive (42) 292-64501 assays (WAKO-Chemicals). The human EZBRAIN42 assay (Millipore), when used for APPSwe and B6.152H mice, gave results comparable to those obtained with the WAKO assay. Only in APPSwe and B6.152H mice the formic acid fraction resulted in detectable A level.

SDS PAGE and Western blotting

Snap frozen right cortices were homogenized and lysed in RIPA buffer (50 mM Tris, NaCl 150 mM containing 0.5% sodium deoxycholate, 1% Nonidet-P-40, 0.1% SDS, pH 7.5 at RT) containing a protease (Roche) and phosphatase inhibitor cocktail (Roche), centrifuged and the supernatant was quantified by the bicinchoninic acid (BCA) assay (Pierce BCA protein assay, Thermo Scientific). After sample incubation at 99°C (10 min), proteins (50 μg/lane) in Laemmli sample buffer (LSB) (50 mM Tris, 50 mM tricine, 2% SDS, 12% glycerol, 80 mM dithiothreitol, pH 8.25 at RT) were loaded on a 12% (w/v) SDS-polyacrylamide gel electrophoresis (PAGE). Proteins were transferred onto a nitrocellulose membrane (Hybond ECL, Amersham).

Membranes were saturated 1 hour at RT with a blocking solution containing 5% (w/v) non-fat dry milk (BioRad) in PBS plus Tween-20 [PBS-T; in mM: 140 NaCl, 6.7 KCl, 20 Na_2HPO_4, 4 KH_2PO_4, 0.1% (w/v) Tween-20, pH 7.4], washed three times with PBS-T (15 minutes) and incubated 24 hours at 4°C with the following primary antibodies: 1:1000 APP-CT695 anti-APP- , -CTF (rabbit, Invitrogen) and 1:3000 anti-actin (mouse, Sigma-Aldrich) as loading control. The membranes were washed three times with PBS-T and incubated with secondary antibody anti rabbit (1:5000) or anti mouse (1:3000) HRP-conjugated. Upon washing with PBS-T, the immunoreactive bands were visualized by enhanced chemiluminescence (Amersham ECL, GE Healthcare Life Science, Pittsburg, US) with a film paper (Amersham Hyperfilm ECL) or with the Nine Alliance software (UVITEC, Eppendorf).

References

1. Fontana, R.; Agostini, M.; Murana, E.; Mahmud, M.; Scremin, E.; Rubega, M.; Sparacino, G.; Vassanelli, S.; Fasolato, C. Early hippocampal hyperexcitability in PS2APP mice: Role of mutant PS2 and APP. Neurobiol. Aging 2017, 50, 64–76.
2. Penny, W.D.; Duzel, E.; Miller, K.J.; Ojemann, J.G. Testing for nested oscillation. J. Neurosci. Methods 2008, 174, 50–61.
3. Rubega, M.; Fontana, R.; Vassanelli, S.; Sparacino, G. A tunable local field potentials computer simulator to assess minimal requirements for phase–amplitude cross-frequency-coupling estimation. Netw. Comput. Neural Syst. 2016, 27, 268–288.
4. Adhikari, A.; Sigurdsson, T.; Topiwala, M.A.; Gordon, J.A. Cross-correlation of instantaneous amplitudes of field potential oscillations: A straightforward method to estimate the directionality and lag between brain areas. J. Neurosci. Methods 2010, 191, 191–200.
5. Fontana, R.; Agostini, M.; Murana, E.; Mahmud, M.; Scremin, E.; Rubega, M.; Sparacino, G.; Vassanelli, S.; Fasolato, C. Early hippocampal hyperexcitability in PS2APP mice: Role of mutant PS2 and APP. Neurobiol. Aging 2017, 50, 64–76.

Supplementary Figures

A

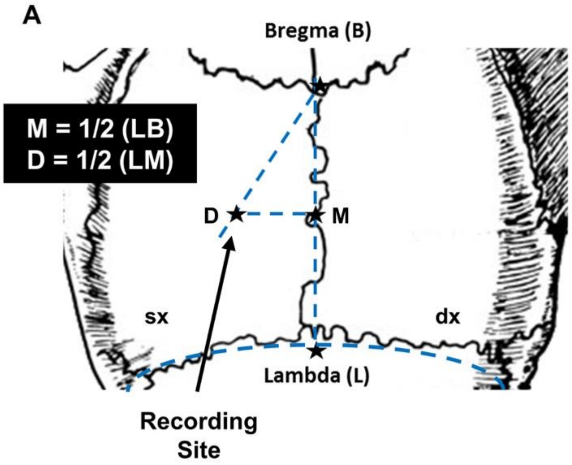

Recording coordinates (mean ± SEM)

	LB (µm)	MD (µm)
**WT 3m**	4564 ± 67	1141 ± 17
**WT 6m**	4568 ± 51	1142 ± 13
**B6.152H 3m**	4468 ± 58	1117 ± 14
**B6.152H 6m**	4544 ± 39	1136 ± 10

B

Anesthesia

Urethane: 1.5g/Kg
Xylazine/Tiletamine-Zolazepam mixture
(Rompun 1 mg/Kg + Zoletil 10mg/Kg)

Administration: *i.p.*

Body Temperature: 37 ± 0.5°C

Physiological condition under anesthesia

(mean ± SEM)

	Heart beat (bpm)	Breath (rpm)
**WT 3m**	231.1 ± 18.8	135.5 ± 6.6
**WT 6m**	288.5 ± 32.3	141.3 ± 7.9
**B6.152H 3m**	277.3 ± 24.1	103.3 ± 12.3
**B6.152H 6m**	270.7 ± 30.5	147.7 ± 13.6

Supp. Fig. 1. Recording coordinates and mouse conditions under anesthesia. (**A**) Procedure used for finding the coordinates for probe insertion, modified from ref. 36 (*left panel*). The average coordinates are shown for B6.152H mice, the group with major electrophysiological and histological differences, with respect to age-matched WT mice (*right panel*). (**B**) The average heart and breath rates under anesthesia are shown; bpm, beats per minute; rpm, respirations per minute. Values were calculated in the windows selected for the analyses, as described in Materials and Methods. No statistical significant difference was found (non-parametric Kruskal-Wallis test).

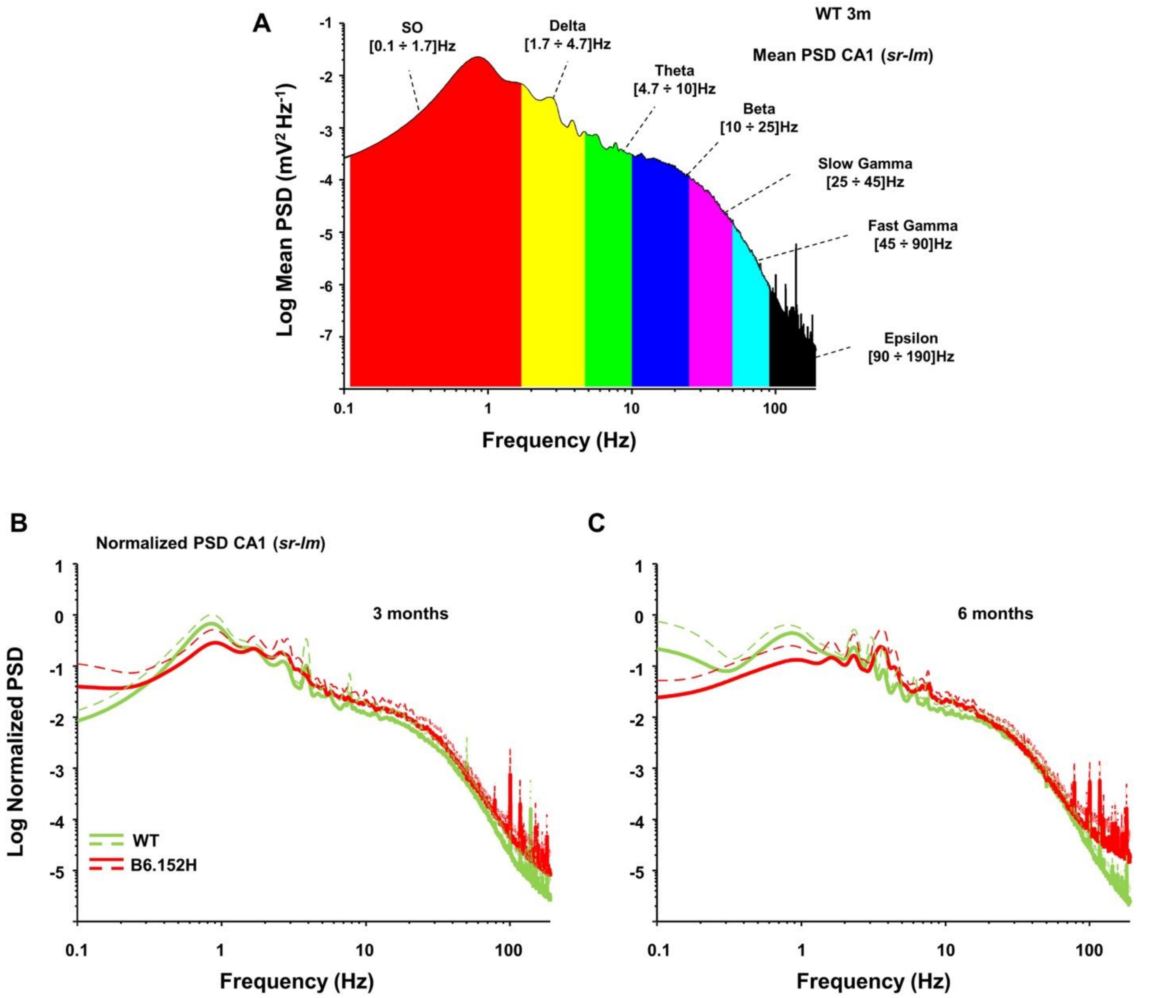

Supp. Fig. 2. Power Spectral Density plots and frequency ranges. Mean PSD plots obtained from recordings in CA1 (*sr-lm*,1500 μm) of a 3-month-old WT mouse, plotted in a log-log scale, showing the frequency ranges used for power analysis (**A**). For comparison of relative power changes, the PSD function was normalized to the total power measured at the same level, for each mouse and averaged for WT and B6.152H mice at 3 (**B**) and 6 months of age (**C**).

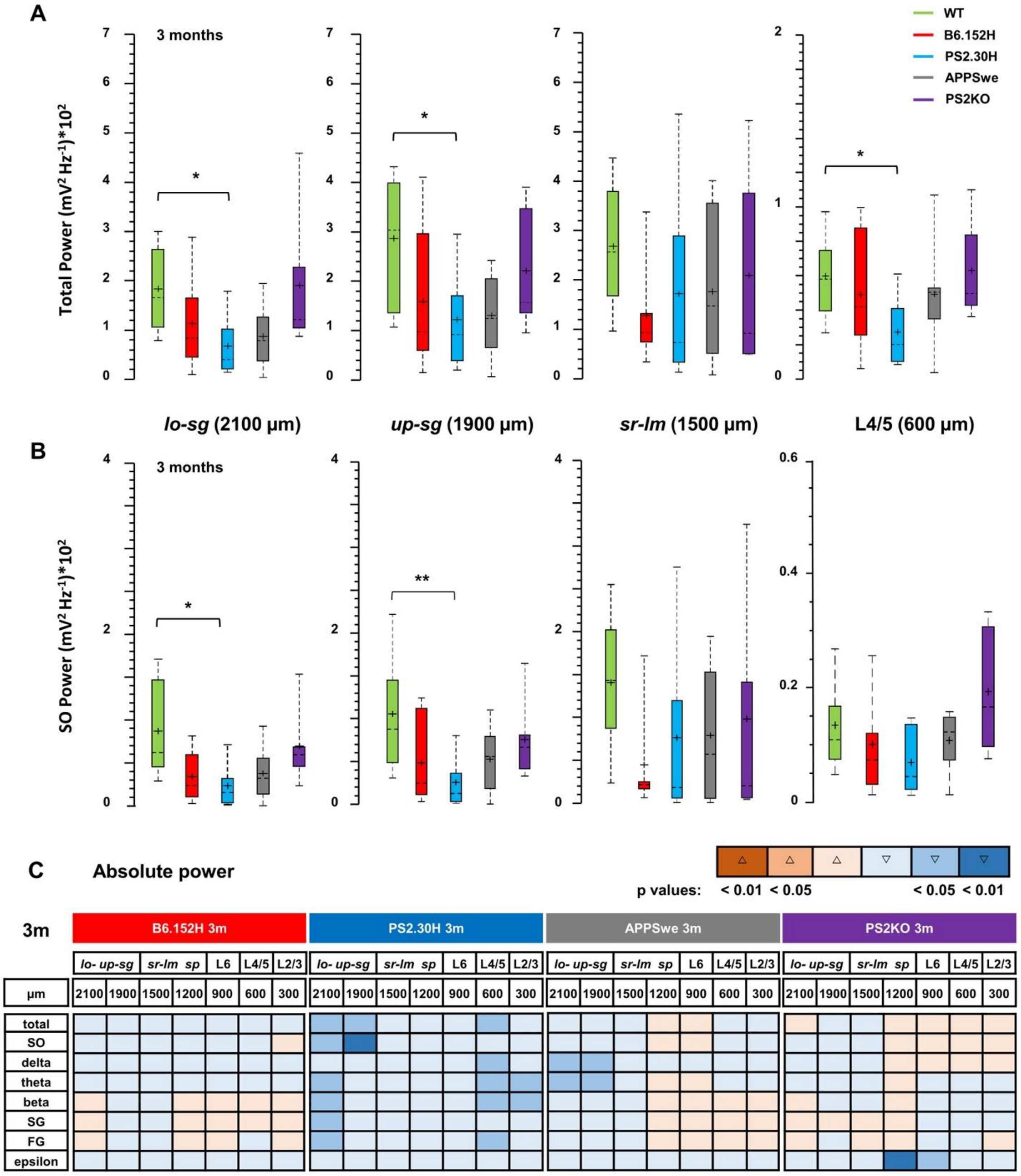

Supp. Fig. 3. Genotype comparison of changes in the absolute power at 3 months of age. Boxplots of the absolute total (**A**) and SO (**B**) powers for all the genotypes at 3 months of age at 2100, 1900, 1500 and 600 µm. Synoptic view of the changes occurring in the absolute total and band powers, at the different depths, for all the genotypes at 3 months of age (**C**). Changes are color-coded in terms of increase or decrease and statistical significance.

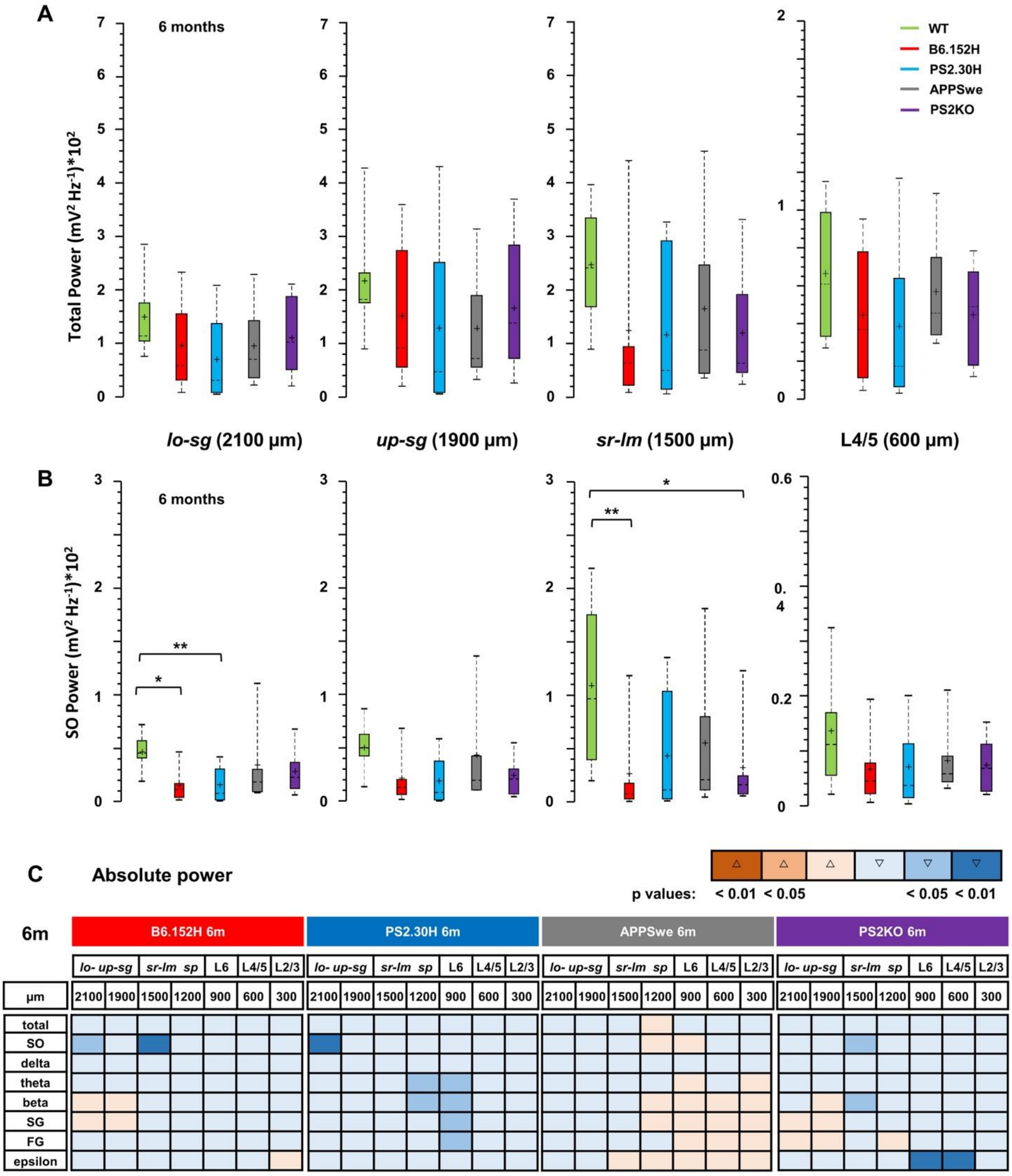

Supp. Fig. 4. **Genotype comparison of changes in the absolute power at 6 months of age.** Boxplots of the absolute total (**A**) and SO (**B**) powers for all the genotypes at 6 months of age at 2100, 1900, 1500 and 600 µm. Synoptic view of the changes occurring in the absolute total and band powers, at the different depths, for all the genotypes at 6 months of age (**C**). Changes are color-coded in terms of increase or decrease and statistical significance.

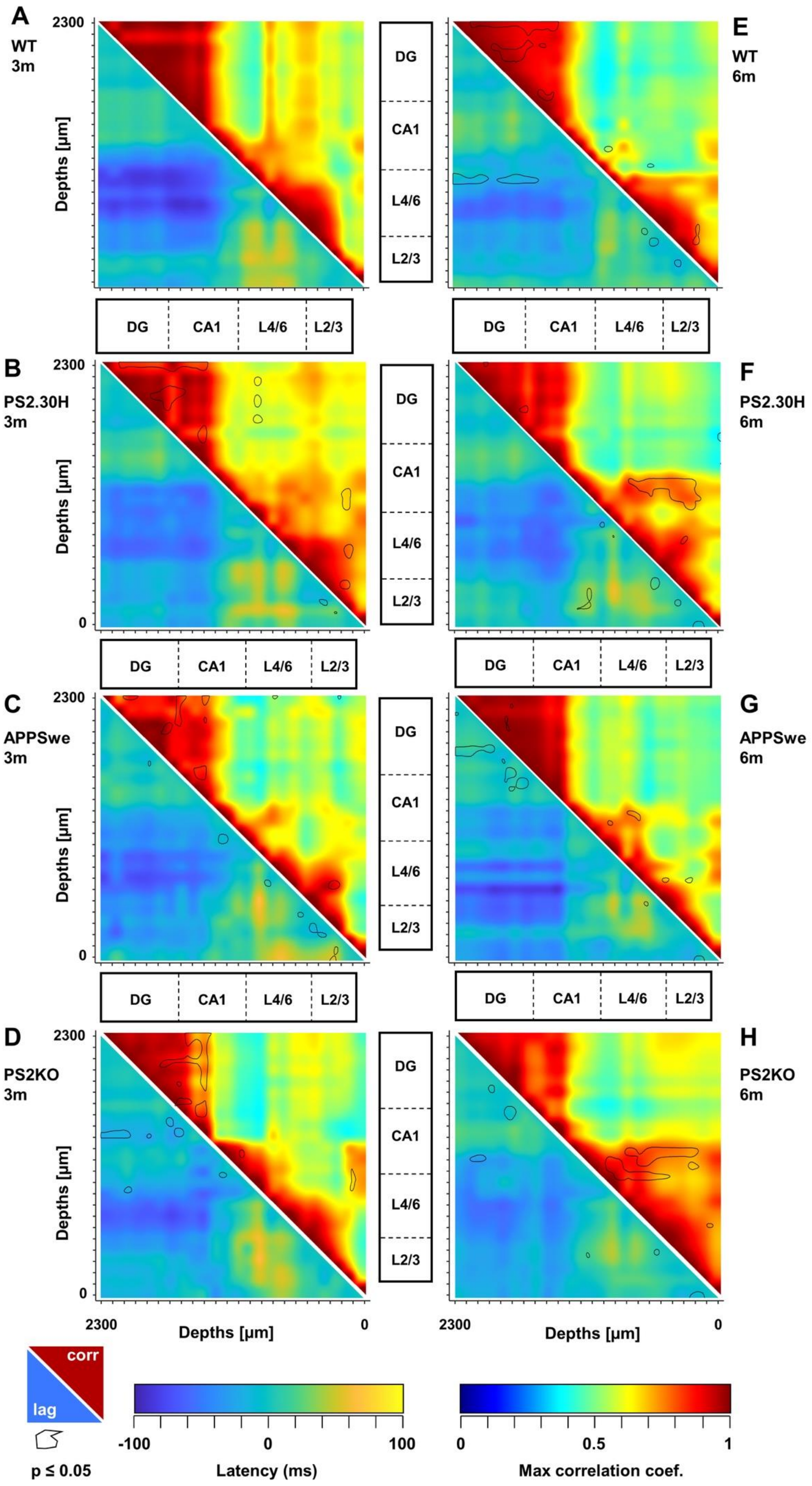
A
WT
3m
B
PS2.30H
3m
C
APPSwe
3m
D
PS2KO
3m
E
WT
6m
F
PS2.30H
6m
G
APPSwe
6m
H
PS2KO
6m
2300
0
Depths [µm]
DG
CA1
L4/6
L2/3
corr
lag
p ≤ 0.05
-100
0
100
Latency (ms)
0
0.5
1
Max correlation coef.

Supp. Fig. 5. Comparison of SO cortico-hippocampal connectivity in tg lines. Matrices of cross-correlation coefficients (upper right) and latencies (lower left) for WT (**A, E**), PS2.30H (**B, F**), APPSwe **(C, G)** and PS2KO (**D, H**) at 3 and 6 months of age, respectively, see Fig. 5 for details. Panels A, E are the same shown in Figure 5.

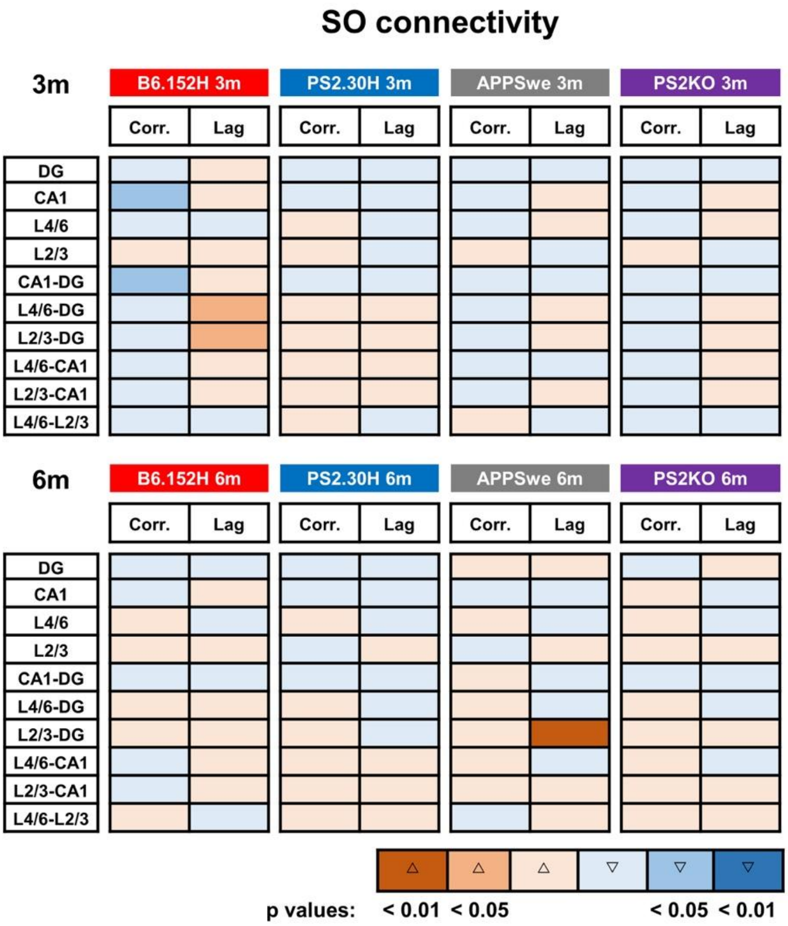

Supp. Fig. 6. Age and genotype comparison of changes in SO connectivity. Synoptic views of the changes occurring in the SO maximal cross-correlations and latencies for all the genotypes at 3 (*upper panel*) and 6 (*lower panel*) months of age with respect to age-matched WT mice. Changes are color-coded in terms of increase or decrease and statistical significance. For quantitative analyses of the regional changes, the cross-correlation coefficients and latencies of each mouse were averaged within (intra-regional) and between (cross-regional) regions, according to the scheme provided in Fig. 6A and then averaged by age and genotype.

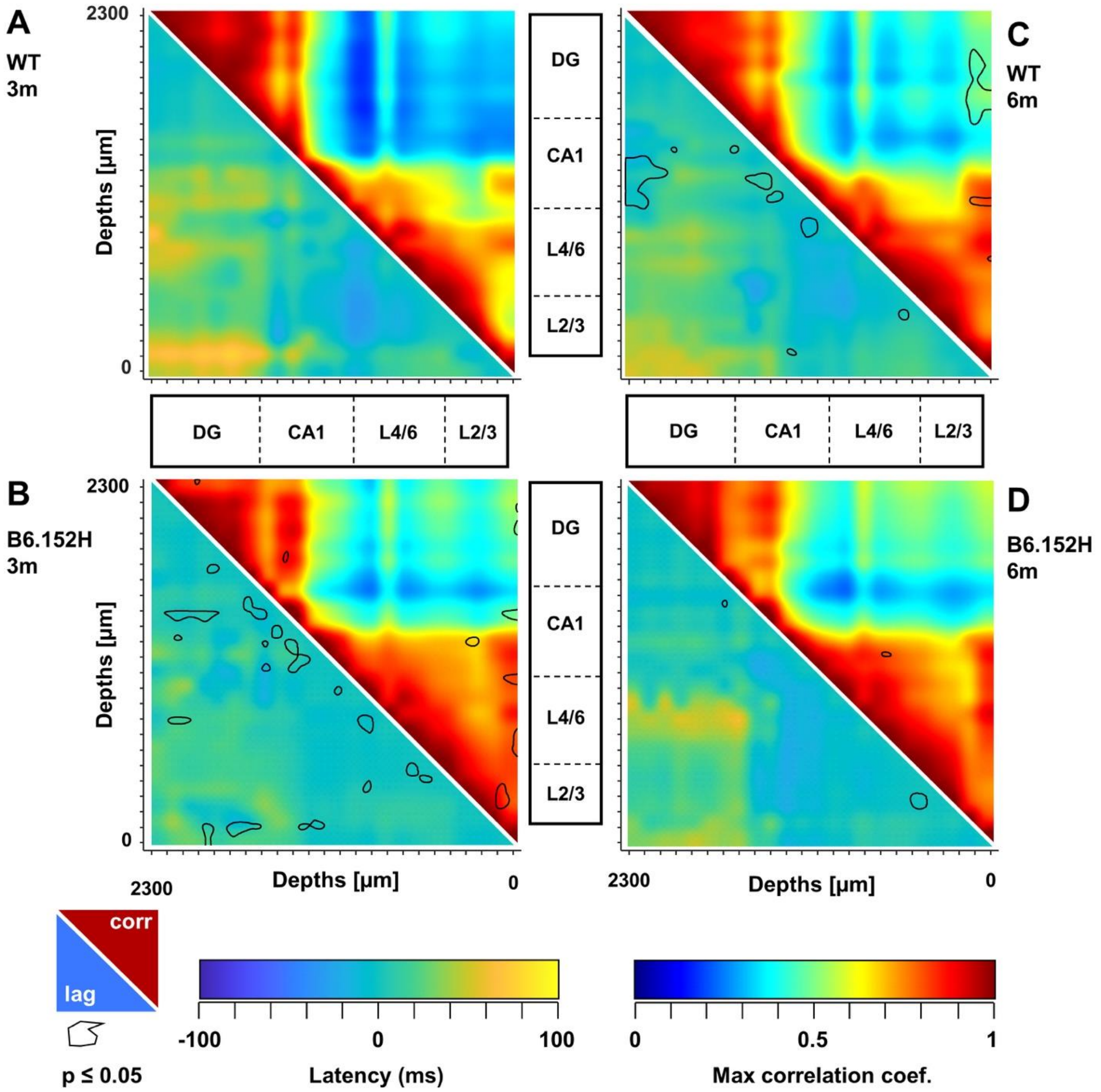

Supp. Fig. 7. Delta cortico-hippocampal connectivity in B6.152H mice. Matrices of cross-correlation coefficients (upper right) and latencies (lower left) for WT (**A, C**) and B6.152H (**B, D**) at 3 and 6 months of age, respectively. See Supp. Fig. 6 for details.

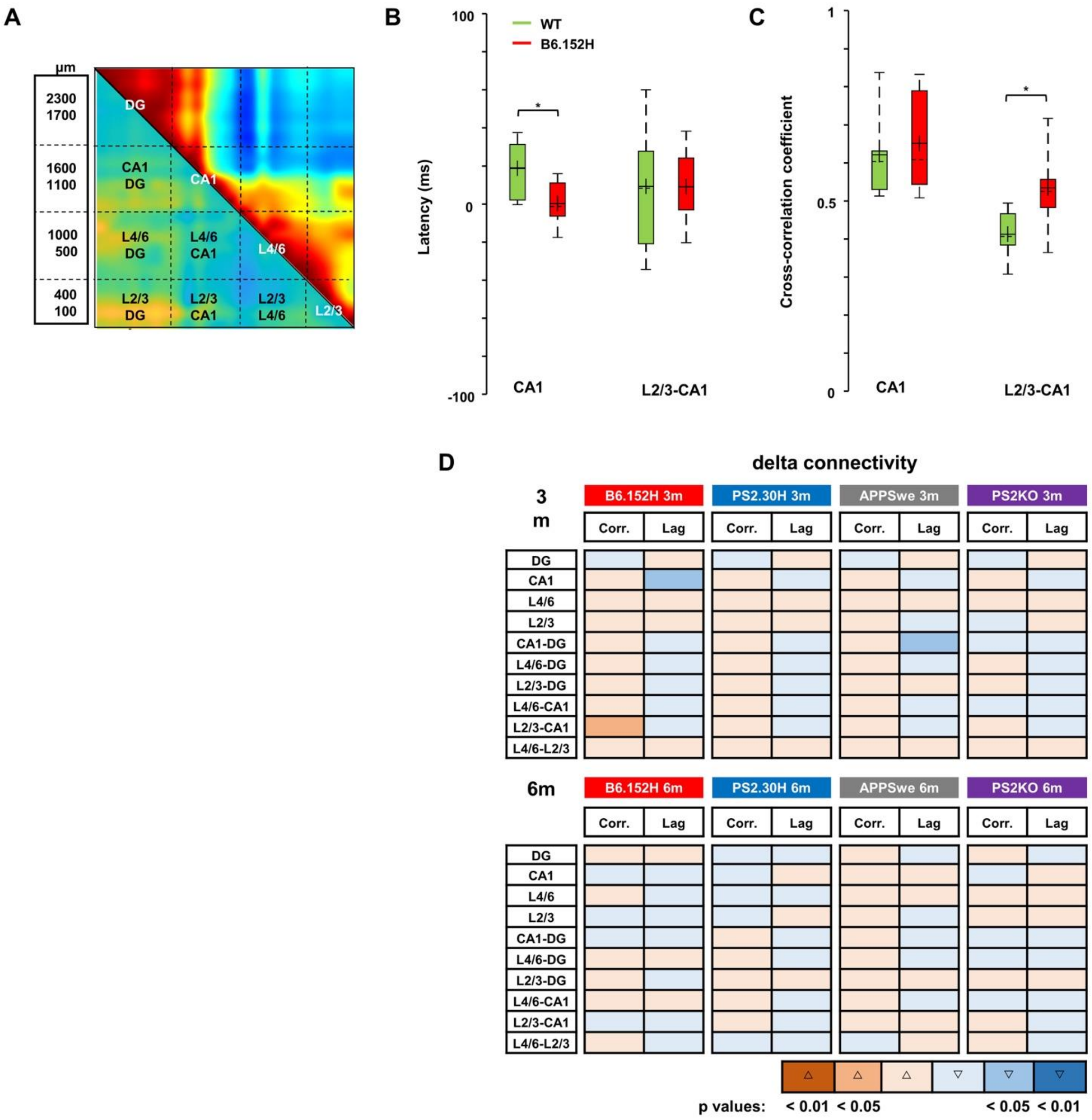

Supp. Fig. 8. Delta cortico-hippocampal correlation and latency changes in tg mice. For quantitative analyses of regional changes, delta maximal cross-correlation coefficients and latencies of each mouse were averaged within (intra-regional) and between (cross-regional) regions, according to the scheme overlaid to the matrices of the 3-month-old WT mice (**A**). Values from were then averaged by age and genotype and shown in boxplots for WT and B6.152H mice as cross-regional latencies (**B**) and cross-correlation coefficients (**C**). In B6.152H mice, delta cortico-hippocampal cross-correlation was increased between L2/3 and CA1, whereas delta latency in CA1 was reduced. (**D**) Synoptic views of the changes occurring in the delta maximal cross-correlations and latencies for all the genotypes at 3 (*upper panel*) and 6 (*lower panel*) months of age with respect to age-matched WT mice.

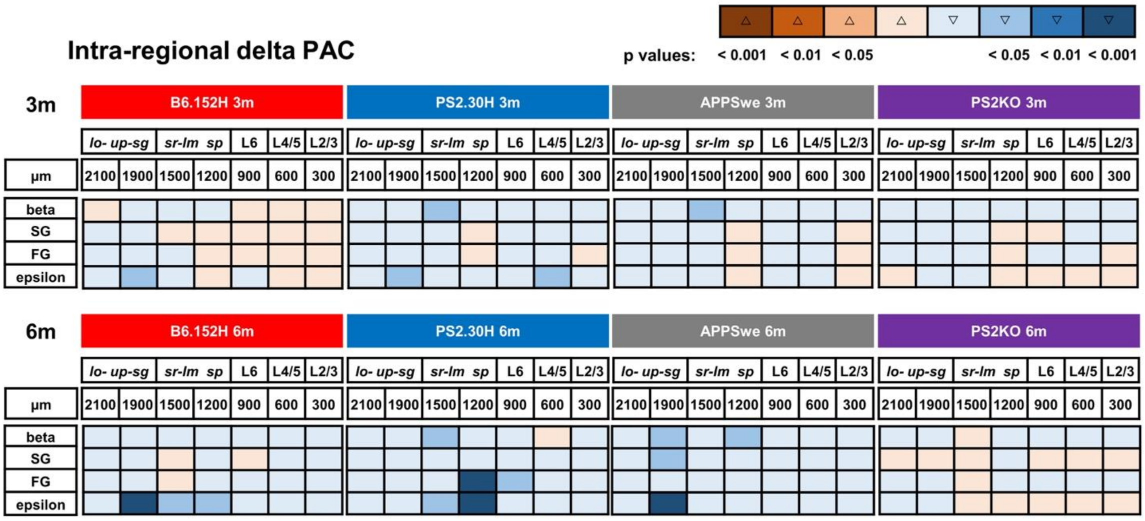

Supp. Fig. 9. Reduced delta Phase-Amplitude Coupling in AD mice. PAC between delta and higher frequencies was measured by the GLM index as described in Supplementary Material. The intra-regional PAC was measured between delta and higher frequencies at each depth. Changes were color-coded in terms of increase or decrease and statistical significance with respect to 3- (*upper panel*) and 6- (*lower panel*) month-old WT mice.
